# Prediction of Vagal Nerve Stimulation Efficacy in Drug-Resistant Epilepsy (PRECISE): Prospective Study for Pre-implantation Prediction/Study Design

**DOI:** 10.3389/fneur.2022.839163

**Published:** 2022-03-21

**Authors:** Irena Dolezalova, Eva Koritakova, Lenka Souckova, Jan Chrastina, Jan Chladek, Radka Stepanova, Milan Brazdil

**Affiliations:** ^1^The First Department of Neurology, Faculty of Medicine, Masaryk University and St. Anne's University Hospital, Brno, Czechia; ^2^Faculty of Medicine, Institute of Biostatistics and Analyses, Masaryk University, Brno, Czechia; ^3^International Clinical Research Center, St. Anne's University Hospital, Brno, Czechia; ^4^Department of Pharmacology, Faculty of Medicine, Masaryk University, Brno, Czechia; ^5^Department of Neurosurgery, Faculty of Medicine, Masaryk University and St. Anne's University Hospital, Brno, Czechia; ^6^Behavioral and Social Neuroscience Research Group, Central European Institute of Technology, Masaryk University, Brno, Czechia; ^7^Institute of Scientific Instruments of the Czech Academy of Sciences, Brno, Czechia

**Keywords:** drug-resistant epilepsy, vagal nerve stimulation, efficacy prediction, statistical model, study design

## Abstract

**Background:**

Vagal nerve stimulation (VNS) can be indicated in patients with drug-resistant epilepsy, who are not eligible for resective epilepsy surgery. In VNS therapy, the responder rate (i.e., percentage of subjects experiencing ≥50% seizure reduction) is ~50%. At the moment, there is no widely-accepted possibility to predict VNS efficacy in a particular patient based on pre-implantation data, which can lead to unnecessary surgery and improper allocation of financial resources. The principal aim of PRediction of vagal nerve stimulation EfficaCy In drug-reSistant Epilepsy (PRECISE) study is to verify the predictability of VNS efficacy by analysis of pre-implantation routine electroencephalogram (EEG).

**Methods:**

PRECISE is designed as a prospective multicentric study in which patients indicated to VNS therapy will be recruited. Patients will be classified as predicted responders vs. predicted non-responders using pre-implantation EEG analyses. After the first and second year of the study, the real-life outcome (responder vs. non-responder) will be determined. The real-life outcome and predicted outcome will be compared in terms of accuracy, specificity, and sensitivity. In the meantime, the patients will be managed according to the best clinical practice to obtain the best therapeutic response. The primary endpoint will be the accuracy of the statistical model for prediction of response to VNS therapy in terms of responders and non-responders. The secondary endpoint will be the quantification of differences in EEG power spectra (Relative Mean Power, %) between real-life responders and real-life non-responders to VNS therapy in drug-resistant epilepsy and the sensitivity and specificity of the model.

**Discussion:**

PRECISE relies on the results of our previous work, through which we developed a statistical classifier for VNS response (responders vs. non-responders) based on differences in EEG power spectra dynamics (Pre-X-Stim).

**Trial Registration:**

www.ClinicalTrials.gov, identifier: NCT04935567.

## Introduction

### Background and Rationale

The main goal of epilepsy treatment is long-term seizure cessation. In two-thirds of patients, seizure-freedom can be established with antiseizure medication (ASM) intake (1). Seizures persist despite ASM treatment in one-third of patients, and these are labeled as drug-resistant or refractory epilepsy. Resective surgery should be considered in all drug-resistant cases. If the patient is not eligible for resection, neurostimulation can be indicated as a therapeutic option.

Vagal nerve stimulation (VNS) is the most commonly used neurostimulation method in the treatment of patients with epilepsy. More than 100,000 patients have already been implanted world-wide. The therapy with VNS provides a high chance for a substantial seizure reduction. Approximately 50% of implanted patients are responders (seizure reduction ≥50%) after 1 year of VNS, and about 5 % of implanted patients reach complete seizure freedom. However, there is a group of implanted patients, non-responders (seizure reduction <50%), who do not significantly benefit from VNS (2–5).

Currently, we are not able to reliably predict the response to VNS based on patients' pre-implantation data. This prediction is crucial for proper selection of VNS candidates, and reduction of unnecessary surgery and costs associated with the implantation. Moreover, the ability to predict VNS efficacy seems to be essential for VNS use in resource-limited countries.

Our group developed and validated a statistical model (Pre-X-Stim) for the prediction of VNS efficacy (6). This algorithm is based on the analysis of routine scalp electroencephalogram (EEG) data, which is the main advantage of our approach because it is an inexpensive, easily-accessible method with minimal burden on the patient. When summarizing the procedure in brief, we retrospectively identified pre-implantation routine scalp EEG recordings in patients treated with VNS for drug-resistant epilepsy. The patients were characterized as responders or non-responders based on the long-term outcomes. The EEG recordings were segmented into several time intervals. In each interval, the changes of dynamics in EEG power spectra were calculated. Based on these changes, the statistical classifier for a prediction of VNS efficacy was developed (Patent No. EP 3437692). The validity of the model was verified on an independent data set, in which it was able to predict the response to VNS with 86% accuracy, 83% sensitivity, and 90% specificity. The usability of this model with different EEG systems was subsequently proven (7). Our previous studies were, however, retrospective and monocentric, and these are the crucial limitations that we would like to overcome with the proposed project, PRECISE (PRediction of vagal nerve stimulation EfficaCy In drug-reSistant Epilepsy).

### Objectives

The study is designed to determine the availability of VNS prediction based on pre-implantation data. The main goal is to validate Pre-X-Stim in a real-life prospective study.

The primary aim of our study is to evaluate whether we are able to predict VNS efficacy in an individual patient based on the pre-implantation scalp EEG. We defined two hypotheses:

1. We hypothesize that there are differences in the dynamics of EEG power spectra in response to activation methods (eye-opening/closing, photic stimulation, hyperventilation) between responders and non-responders to VNS therapy.
2. We hypothesize that we can predict the patient's response to VNS therapy in terms of responder vs. non-responder by our statistical model based on the changes in the dynamics of EEG power spectra.

Based on these hypotheses the primary and secondary endpoints were established. Primary endpoint involves the accuracy of the statistical model for prediction of response to VNS therapy in terms of responders and non-responders in drug-resistant epilepsy, evaluated after 2 years. Secondary endpoints include (1) the quantification of differences in EEG power spectra (Relative Mean Power, %) between real-life responders and real-life non-responders to VNS therapy in drug-resistant epilepsy; (2) prediction of patients' response to VNS therapy in terms of responders (≥50% seizure reduction from baseline) and non-responders (<50% seizure reduction from baseline). Each endpoint will be evaluated 2 years after recruitment.

### Trial Design

PRECISE is an investigator-initiated study. It is a prospective multicentric, multinational clinical study that will be started in January 2022. The patients with drug-resistant epilepsy will undergo standard VNS implantation based on a clinical decision. Before implantation, a scalp EEG, according to the study protocol, will be recorded. The EEG will be mathematically processed, and the patient's predicted outcome in terms of predicted responder vs. predicted non-responders will be statistically determined. The stimulation parameters and ASM therapy will be modified according to the best clinical practice. The real-life outcome will be measured by the percent of seizure reduction. Subsequently, the real-life and predicted outcomes will be compared in terms of accuracy, sensitivity, and specificity.

The proposed study is based on the results of our previous work, through which a statistical model was developed for the prediction of VNS efficacy based on a mathematical and statistical analysis of scalp EEG data (6).

## Methods and Analysis

### Study Setting

The PRECISE study has not yet been recruited. The study is planned to be initiated in nine European countries (Czech Republic, France, Germany, UK, Spain, Austria, Portugal, Italy, and Belgium) in 2022.

Brno Epilepsy Center, the coordinating center for the study, is responsible for education and training of research staff, tracking participants' enrolment, mathematical analysis, including statistics of scalp EEG, and reporting of the study.

The other collaboration centers are responsible for patient selection according to eligibility criteria, adherence to all criteria of the study as described below, and adjustment of ASM and VNS therapy according to the best clinical practice.

### Eligibility Criteria

Inclusion criteria include a diagnosis of drug-resistant epilepsy based on the definition of the International League against Epilepsy (8). The patient has to be indicated to VNS therapy based on a clinical decision.

All adult patients with drug-resistant epilepsy indicated to VNS will be screened in participating centers for inclusion criteria. Written informed consent for each study participant will be obtained prior to any data collection.

Inclusion criteria are as follows:

1. Drug-resistant epilepsy indicated to VNS based on clinical decision. Drug-resistant epilepsy is defined as a failure of adequate trials of two tolerated, appropriately chosen and used antiepileptic drug schedules (whether as monotherapy or in combination) to achieve sustained seizure freedom (8).
2. Age ≥18 years.
3. Availability to record 20 min EEG with photic stimulation, hyperventilation, and eye-opening/closing according to the pre-defined protocol.
4. Availability of seizure diaries at least 3 months before VNS implantation OR reliable information about seizure frequency at least 3 months before VNS implantation.
5. The ability of a patient/family member/caregiver to record seizures precisely into seizure diaries OR the ability of a patient/family member/caregiver to report seizures precisely in other ways.
6. In cases with very high seizure frequency (several seizures per day), it is acceptable to report only the days without any seizures.

Patients meeting any of the following criteria are excluded:

1. The indication and planning of resective brain surgery as a treatment option for drug-resistant epilepsy. If a patient clearly demonstrates refusal of resective surgery, the patient can be included in the study.
2. The presence of psychogenic non-epileptic seizures which cannot be reliably distinguished from epileptic seizures by a patient/family member/caregiver.
3. The presence of another condition that can resemble epileptic seizures and which cannot be reliably distinguished from epileptic seizures by a patient/family member/caregiver.
4. Metabolic condition or other diseases, in which an increase of seizure frequency can be expected.
5. The inability of a patient to make regular visits.
6. Life expectancy shorter than 2 years.

### Who Will Take Informed Consent?

The study protocol including the informed consent will be reviewed and approved by the Institutional Review Board at each of the sites. A trained study investigator will describe the study to patients or to authorized surrogates if applicable. Patients will also receive information sheets. The study investigator will discuss the study with patients based on the information provided in the information sheets. The study investigator will obtain the written informed consent from patients willing to participate in the trial. In the case of patients who are unable to give consent because of a medical condition, their ability to participate in the study will be assessed by a medical council consisting of at least one independent physician, who is informed about the study details, and one study investigator.

### Additional Consent Provisions for Collection and Use of Participant Data and Biological Specimens

A part of informed consent will be dedicated to reusing the data obtained in the proposed study to improve the proposed statistical model or the development of a novel predictive statistical model. The study conforms to the Consolidated Standards of Reporting Trials (CONSORT) guidelines.

### Interventions

#### Explanation for the Choice of Comparators

Not applicable. The comparator is not used in this study.

#### Intervention Description

Assessment 0 includes screening for eligibility criteria and obtaining baseline data on subjects participating in the study. After signing the informed consent, the patient will be given a unique code with which all data will be labeled. The demographic information, health history, including psychiatric disease and type of epilepsy, concomitant medication (ASM and other drugs), and seizure frequency will be collected. The seizure frequency will be analyzed based on the patient diary. Standard EEG will be recorded according to the protocol described below. Patients will be asked to fill in a questionnaire focusing on the quality of life.

### Protocol for EEG Recording

The EEG will be recorded on the EEG recording system with a sampling frequency of 250 Hz or higher, with electrodes placed on the scalp according to the 10–20 system (with the following electrodes: Fp1, Fp2, F7, F3, Fz, F4, F8, T3, C3, Cz, C4, T4, T5, P3, Pz, P4, T6, O1, and O2). Different electrode names are allowed, but their position must correspond with the position of the previously defined electrodes. Additional electrodes are also allowed, but will not be evaluated within the study. EEGs with fewer or differently-positioned electrodes are not allowed.

The EEG is recorded in a supine position with eyes closed, except for periods with opening of eyes. The recorded EEG will have a duration of 20 min or more and must contain defined segments in the following order as in Table 1.

**Table 1:** Predefined segments ordering in the course of EEG recording.

Segment 1	Rest 1	duration at least 2 min
Segment 2	Eyes opening/closing	duration at least 10 s
Segment 3	Rest 2	duration at least 10 s; immediately after eye closure
Segment 4	Photic stimulation	stimulation frequencies: 5 Hz (10 s)−10 Hz (10 s)−15 Hz (10 s)−20 Hz (10 s)−25 Hz (10 s)−30 Hz (10 s)−25 Hz (5 s)−20 Hz (5 s)−15 Hz (5 s)−10 Hz (5 s)−5 Hz (5 s); light intensity at least 0.7 Joule
Segment 5	Hyperventilation	duration at least 4 min (2 min hyperventilation by nose + 2 min hyperventilation by mouth)
Segment 6	Eyes opening/closing	duration at least 10 s
Segment 7	Rest 3	duration at least 10 s, immediately after eye closure
Segment 8	Rest 4	duration at least 2 min

The recorded EEG will be anonymized and labeled by a unique code previously attributed to a patient. In the next step, the EEG will be sent for mathematical/statistical processing. Based on this processing, the patients' predicted response to VNS will be determined in terms of predicted responders (seizure reduction ≥50%) vs. predicted non-responders (seizure reduction <50%). Neither the patient nor the physician will be informed about the classification results.

Two assessments are designed for patients' follow-up:

Assessment 1−1 year after stimulation initialization, and

Assessment 2−2 years after stimulation initialization.

The patients will be evaluated 1 and 2 years after the start of VNS therapy. The patient will be requested to report any changes in health history, including psychiatric disease, and specific requests will be focused on changes in medication (ASM and other drugs), VNS parameters settings, and seizures. The patients will be asked whether unexpected events influenced their clinical response. If these unexpected events are regarded as “clinically highly important” (such as non-compliance or other newly-diagnosed disease worsening epilepsy), the patient can be excluded based on clinical decision. The data on seizure frequency will be collected by reviewing the patient's diary. The reported seizure frequency will be compared with pre-implantation seizure frequency, and real-life response will be calculated in terms of real-life responders (seizure reduction ≥50%) or real-life non-responder (seizure reduction <50%). The patients will fill in a questionnaire focusing on the quality of life. If the patient does not tolerate at least the minimal VNS settings (0.75 mA) because of serious adverse events, they will be excluded from further analysis (the patient's data will be reported separately). The patients included in our study will be managed according to the best clinical praxis, i.e., the change of ASM and VNS parameters will be done based on the clinicians' decision to reflect the real-life response.

The information collected in Assessments 1 and 2, including real-life response to VNS, will be labeled with the patient's unique code and sent to the coordinating center, where it will be compared with the patient's predicted response.

At the end of Assessment 2, patients will be asked if they are interested in the predicted response to VNS. If the answer is yes, the study physician will contact the coordinating center, where the predicted response for a given patient will be conveyed. The patient will be informed about the predicted response by telephone or during a regular visit (not a part of the study protocol, described below).

### Criteria for Discontinuing or Modifying Allocated Interventions

If a patient does not tolerate at least the minimal VNS setting (0.75 mA) because of serious adverse events, the patient will be excluded from further analysis (the patient's data will be reported separately).

### Strategies to Improve Adherence to Interventions

Training before initialization will ensure the fidelity during the whole study. If there are doubts, the coordinating center will solve them.

### Relevant Concomitant Care Permitted or Prohibited During the Trial

Regular visits—not part of the study protocol.

There are regular visits between Assessments 0, 1, and 2. The regular visits are not part of the study protocol. The physician will adjust the VNS parameters and change concomitant ASM according to the best clinical practice to obtain the best response with minimal side-effects. The regular visits will be scheduled according to the individual habits of cooperating centers. The change of ASM (types and dosing) is allowed within the whole study.

### Outcomes

The primary outcome of the study is the accuracy of the statistical model for prediction of response to VNS therapy in drug-resistant epilepsy, expressed as a percentage of correct predictions (responders vs. non-responders), evaluated after 2 years.

The secondary outcomes of the study are the quantification of differences in EEG power spectra (Relative Mean Power, %) between real-life responders and real-life non-responders to VNS therapy in drug-resistant epilepsy and the prediction of patients' response to VNS therapy in terms of responders, expressed as sensitivity of the model, and non-responders expressed as specificity of the model. If possible, these data can be used for further improvement of the statistic model.

The study is concerned with the verification of the real-life validity of our statistical classifier for the prediction of VNS response based on pre-implantation EEG data. The patients will be classified based on their predicted response as predicted responders (≥50% seizure reduction) and predicted non-responders (<50% seizure reduction). In Assessments 1 and 2, respectively, the patients' real-life response will be determined: real-life responders (≥50% seizure reduction) vs. real-life non-responder (<50% seizure-reduction). The predicted and real-life responses will be statistically compared in terms of accuracy, sensitivity, and specificity.

The individual patients' groups (predicted responders vs. predicted non-responders, real-life responders vs. real-life non-responders) will be compared based on their demographic data, health history, ASM, and VNS parameters. A suitable statistical test will be chosen concerning the characteristics of the data (Pearson's Chi-squared test for categorical variables, and Wilcoxon-Mann-Whitney test for continuous variables).

### Participant Timeline

The schedule of enrolment, intervention and visits is summarized in Table 2. The study is focused on patients indicated to VNS therapy as their standard for clinical care and is concerned with the prediction of VNS efficacy based on pre-implantation EEG. The duration of the study participation in a given patient is 2 years.

**Table 2:** Participant timeline.

	**Assessment 0**	**Assessment 1**	**Assessment 2**
	**Day 0**	**Year 1**	**Year 2**
Screening—inclusion/exclusion criteria	X		
ICF	X		
Demographic information	X	X	X
Health history	X	X	X
Concomitant medication	X	X	X
Seizure frequency	X	X	X
EEG	X		

*Between assessment 0 and Assessment 1, VNS will be performed, but it is not a part of this study*.

### Sample Size

The sample size was estimated for the primary endpoint, which was defined as the accuracy of the statistical model for prediction of response to VNS therapy in terms of responders and non-responders in drug-resistant epilepsy. During the verification of the validity on the independent dataset, the model was able to predict the response to VNS with 86% accuracy. The minimal clinically significant accuracy was set to 70%. The sample size is estimated for the following hypothesis:

Hypothesis H0: *p* = 0.7 vs. Hypothesis H1: *p-value* > 0.7.

At the level of significance α = 0.05 and with a power of 90%, the number of patients needed for the analysis of the primary endpoint is 57. Assuming a drop-out rate of 50%, a total number of 114 patients should be enrolled.

### Recruitment

Recruitment of the patients is planned from January 2022. During the first 2 years, the total planned number of 140 patients will be recruited. The patients will be recruited within cooperating epilepsy centers. The recruitment limited to cooperating centers will ensure the correct indication for VNS therapy and the adjustment of VNS parameters according to the best medical practice, which are not part of the study protocol itself, but are crucial for the study results.

The VNS will be indicated in a particular patient based on a clinical decision as a standard of the patient's therapy (i.e., the indication of VNS is not a part of the proposed project). The patient will be informed about the study on a pre-implantation visit or by a telephone call, during which initial eligibility for the study will be determined, and the study protocol and design will be explained by the research staff.

### Assignment of Interventions: Allocation

#### Sequence Generation

Not applicable. This study is not randomized.

#### Concealment Mechanism

Not applicable. This study is not randomized.

#### Implementation

The study investigator enrolls the participants. The other collaboration centers are responsible for patients' selection according to eligibility criteria, adherence to all criteria of study as described below, and adjustment of ASM and VNS therapy according to the best clinical practice, and enrolling the participants.

### Assignment of Interventions: Blinding

#### Who Will Be Blinded?

Not applicable. This study is not blinded.

#### Procedure for Unblinding If Needed

Not applicable. This study is not blinded.

### Data Collection and Management

#### Plans for Assessment and Collection of Outcomes

The study will collect demographic and baseline characteristics from medical records and electronic medical records, including age, sex, type of admission, and baseline characteristics such as body weight, body height, patient history, and pharmacological history. Results of EEG tests and neurological assessment will be documented in the electronic medical records and entered by the trial investigator in the electronic case report form (eCRF).

#### Plans to Promote Participant Retention and Complete Follow-Up

The study site will make every reasonable effort to follow the participant for the entire study period. Study site staff members will be responsible for developing and implementing local standard operating procedures to achieve maximal follow-up.

#### Data Management

The study data will be entered online in an Internet-based database (RedCap®) and collected from medical records. The study staff members will have access to the medical records of the patients. Investigators will be responsible for screening the patients, obtaining informed consent, collecting study data, and entering the data in the eCRF. A statistician will analyse the study data in cooperation with a principal investigator. The data will be stored for 15 years after completion of the study and then destroyed. To promote data quality, the eCRFs of each participant will be reviewed by another member of the study team as monitor.

#### Confidentiality

All study-related information will be stored securely at the study site. All participant information will be stored in locked file cabinets in areas with limited access. All local databases will be secured with password-protected access systems. Forms, lists, logbooks, appointment books, and any other listings that link participant ID numbers to additional identifying information will be stored in a separate locked file in an area with limited access.

### Plans for Collection, Laboratory Evaluation, and Storage of Biological Specimens for Genetic or Molecular Analysis in This Trial/Future Use

Not applicable. In this study, no biological specimens for genetic or molecular analysis will be collected.

### Statistical Methods

#### Statistical Methods for Primary and Secondary Outcomes

Subjects will be classified using logistic regression or another classifier (such as support vector machines or linear discriminant analysis) as predicted responders or non-responders. The predicted outcomes will be compared with real-life outcomes to determine the accuracy, sensitivity, and specificity of our statistical model. The accuracy will be calculated as the proportion of correctly predicted responders and correctly predicted non-responders within the whole analyzed population. The minimum clinically significant accuracy was set to 70%. The sensitivity of the model will be expressed as the proportion of predicted responders within all real-life responders. The specificity of the model will be expressed as the proportion of predicted non-responders within all real-life non-responders.

Differences in the EEG power spectra between real-world/predicted responders vs. non-responders will be examined using two-sample *t*-test or Mann-Whitney test, as applicable.

As a level of statistical significance, α = 0.05 will be used for all statistical tests.

#### Interim Analyses

Not applicable. Interim analysis is not planned.

#### Methods for Additional Analyses (e.g. Subgroup Analyses)

Not applicable. Additional analysis is not planned.

#### Methods in Analysis to Handle Protocol Non-adherence and Any Statistical Methods to Handle Missing Data

No analysis populations are defined for this study. Protocol non-adherence will be assessed by the principal investigator case by case. Patients with major protocol deviations will be excluded from the analysis.

Missing data are not planned to be imputed. However, in the event of substantial missing data for any parameter, a sensitivity analysis using any method of imputation could also be used.

#### Plans to Give Access to the Full Protocol, Participant Level-Data, and Statistical Code

Data sharing statement: No later than three years after the collection of the second year after the last patient's last visit, we will deliver a completely anonymized data set to an appropriate data archive for sharing purposes. Statistical codes will be archived following GCP and SOPs.

### Oversight and Monitoring

#### Composition of the Coordinating Center and Trial Steering Committee

Not applicable. No coordinating center or a trial steering committee will be established.

#### Composition of the Data Monitoring Committee, Its Role and Reporting Structure

Not applicable. No data monitoring committee will be established. The study interventions as VNS or EEG are parts of the standard care of patients with drug-resistant epilepsy. The studied tool is a statistical mathematical model predicting the responsiveness to VNS.

#### Adverse Event Reporting

Serious adverse events have been reported according to the standard rules and standard operating procedures of the coordinating unit. All study participants will be monitored for potential adverse events. We do not expect any adverse event to be associated with the Pre-X-Stim because it is a statistical mathematical model evaluated as a tool able to predict the response to VNS.

#### Frequency and Plans for Auditing Trial Conduct

Trial conduct will be audited by the Institutional Ethics Committee every year during the study. The process of auditing will be independent of the investigator and sponsor.

## Discussion

In the population of patients with drug-resistant epilepsy, VNS represents a well-established treatment option to standard ASM and resective surgery. ASM fails to abolish seizures in one-third of patients (1). Resective surgery represents a gold standard for the treatment of these patients with a high probability of complete seizure remission (9). However, there is a large group of patients who are not eligible for resective surgery because of several reasons, namely, our inability to form a rational hypothesis on the precise localization of the epileptogenic zone even with the use of invasive EEG recording, multifocal epilepsy, severe intellectual disability, and personal reasons. In all these cases, VNS can represent a method of choice with a relatively low risk of surgical and post-surgical complications (10).

We have relatively precise knowledge about VNS efficacy in drug-resistant epilepsy based on our experience with a large group of already implanted patients (2–5, 11). An individual patient has a chance of about 50% to become a responder (≥50% seizure reduction) in the first year, and this chance increases to ~60% in the fifth year. The VNS has proved its superiority with respect to seizure-reduction and quality of life, even in a prospective randomized controlled trial (12).

It is widely known that better response to VNS is present in children, in patients with generalized or post-traumatic epilepsy or patients with tuberous sclerosis. However, we are not able to predict the efficacy of an individual patient based on his/her pre-implantation data. In this situation, it is clear that some patients, family members, caregivers, and physicians do not have confidence in this treatment option. It seems crucial to reliably identify responders to VNS based on their clinical data pre-operatively, to minimize unnecessary surgery and additional costs.

At the moment, four studies have focussed on the prediction of VNS efficacy based on pre-implantation data (see below). Remarkably, all works share a similar theoretical concept, specifically the functional mechanism of VNS action. The mechanism of VNS action, despite not being fully understood, relies on the afferent projections of the vagal nerve to the nucleus tractus solitarius (NTS). NTS has a dense connection with other brainstem structures, thalamus, and limbic structures, and just the modulation of thalamocortical circuits is suspected to be responsible for VNS action (13–16).

Our group developed the statistical classifier based on standard EEG records. Moreover, we managed to prove its validity on an independent patient data set. On the other hand, the most important limitation associated with our previous results is the retrospective and monocentric nature of our study. We aim to overcome these limitations through the proposed project PRECISE.

PRECISE will be a prospective multicentric study that will be conducted in co-operation with several well-established and experienced epilepsy centers in Europe. These centers were chosen to be able to correctly identify suitable candidates for VNS therapy, adjust ASM or VNS parameters to obtain the best response, and minimalize side-effects. We decided on this “best clinical practice” model because this approach reflects the regular daily practice that is essential for further applicability of our statistical classifier. This classifier is based on analyzing routine scalp EEG, which we supposed to be its main advantage. The scalp EEG is a painless, non-distressing method that can be recorded everywhere with minimal costs for purchasing the device. Above all, routine EEG recording is an obligatory investigation performed in all patients suffering from drug-resistant epilepsy. Thus, the design of our study is uncomplicated, which is essential for patient recruitment and study fidelity. The patients have to make regular visits for VNS and ASM adjustments. We added three additional assesments—one before and two after stimulation initialization (year one and two). During these visits, the primary clinical information and seizure frequency will be evaluated. The most problematic and discussable part of our protocol is the evaluation of seizure frequency. This activity is standardly done by a review of patients' seizure diaries, which is the practice that is currently used in all clinical studies determining the capability of therapeutic intervention to reduce seizure frequency. We are very much aware that the information in seizure diaries could be influenced both unconsciously (i.e., postictal amnesia) and consciously (i.e., rental tendencies). However, there is currently no available possibility to determine the precise seizure frequency over the course of 2 years. We included only adult patients (i.e. older than 18 years). If we prove the utility of our statistical classifier in this population, we would like to conduct a similar study for children as well, because they represent a population with a high benefit from correctly indicated VNS and form approximately one-third of all implanted patients.

In the text: We also see the limitations of our study. We primarily focused on seizure frequency and seizure reduction. On the other side, it would be helpful to analyse the impact of VNS on other aspects of patients' life, i.e., mood, cognition, and memory. However, these analyses require more time for both physicians and patients. We tried to keep our study as simple as possible because we needed to include a relatively large proportion of those implanted to reach the results in a realistic period of 2–3 years. This limitation is conditioned by the fact that each center implants only a few (5–10) adult patients per year, which was the information obtained in our survey among European epilepsy centers.

As mentioned above, three other studies predicted VNS efficacy on pre-implantation data with similar accuracy (17–19). Ibrahim et al. (17) published a prediction model based on the analysis of resting-state functional magnetic resonance imaging (fMRI) in a group of 21 children with drug-resistant epilepsy. They demonstrated that thalamocortical connectivity to the anterior cingulate and insular cortices is stronger in responders than in non-responders (17). The second study published by Babajani-Feremi et al. (18) predicts VNS efficacy by analysis of resting-state magnetoencephalography (MEG). The authors calculated three global graph measures on recorded resting-state MEG, which include modularity, transitivity, and characteristic path length, in which they found statistically significant differences between responders and non-responders (18). The third study by Mithani et al. (19) proved the differences in white matter microstructures in diffusion tensor imaging (DTI) between responders and non-responders. These differences were subsequently used for the construction of a classifier for VNS response prediction (19).

To conclude, there are several studies published recently that promise to predict VNS response based on available electrophysiological and neuroimaging data. We formulated the statistical model working as a classifier for VNS prediction in retrospective data sets. To determine the validity of this classifier, it needs to be evaluated in a prospective multicentric study. We believe this approach has the potential to significantly improve clinical management of drug-resistant epilepsy patients.

## Ethics and Dissemination

### Ethics Approval and Consent to Participate

This protocol and the template of informed consent form were reviewed and approved by the Ethical Committee of the University Hospital St. Anne's Brno, with respect to scientific content and compliance with applicable research and human subject regulations. Informed consent will be obtained from all participants prior to enrolment in the study.

### Plans for Communicating Important Protocol Amendments to Relevant Parties (e.g., Trial Participants, Ethical Committees)

Any modification of the protocol that may affect the conduct of the study, the potential benefits to the patient, or patient safety, including changes in study objectives, study design, patient population, sample sizes, study procedures, and significant administrative aspects, will require a formal amendment of the protocol. Any amendment will be agreed on and approved by the ethics committee before implementation, and the health authorities will be notified, in accordance with local regulations. The protocol amendment will be sent to all members of the study team by e-mail. All attending investigators and study nurses of the study units will be acquainted with the amendment at a meeting. The invitation to the meeting will be sent by e-mail. The protocol of the study and its amendments will be available at each study unit.

### Dissemination Plans

We intend to submit the results of the study to be published in a peer-reviewed international medical journal.

## Ethics Statement

The studies involving human participants were reviewed and approved by Ethic Committee St. Anne's University Hospital. The patients/participants provided their written informed consent to participate in this study.

## Author Contributions

ID and LS: design of the study, protocol preparation, and manuscript preparation. EK and RS: statistical analysis. JChr: design of the study and protocol. JChl: mathematical analysis. MB: conceived the study and led the proposal and protocol development. All authors contributed to the article and approved the submitted version.

## Funding

This study was supported from the state budget through the Ministry of Education, Youth and Sports by project VI CZECRIN (LM2018128), from the European Regional Development Fund – project CZECRIN_4 PATIENTS (CZ.02.1.01/0.0/0.0/16_013/0001826), and from the Ministry of Health of the Czech Republic, grant number: NV19-04-00343.

## Conflict of Interest

The authors declare that the research was conducted in the absence of any commercial or financial relationships that could be construed as a potential conflict of interest.

## Publisher's Note

All claims expressed in this article are solely those of the authors and do not necessarily represent those of their affiliated organizations, or those of the publisher, the editors and the reviewers. Any product that may be evaluated in this article, or claim that may be made by its manufacturer, is not guaranteed or endorsed by the publisher.
